# Clinical presentation and cardiac imaging findings in patient cases of perimyocarditis and pericarditis with a temporal association to SARS-CoV-2 vaccination

**DOI:** 10.3389/fcvm.2022.964412

**Published:** 2022-08-04

**Authors:** Anders Lehmann Dahl Pedersen, Gregory Wood, Christian Alcaraz Frederiksen, Brian Bridal Løgstrup, Morten Kvistholm Jensen, Lars Østergaard, Won Yong Kim, Steen Hvitfeldt Poulsen

**Affiliations:** ^1^Department of Cardiology, Aarhus University Hospital, Aarhus, Denmark; ^2^Department of Infectious Diseases, Aarhus University Hospital, Aarhus, Denmark

**Keywords:** perimyocarditis, pericarditis, COVID-19 vaccine, cardiovascular magnetic resonance imaging, SARS-CoV-2, echocardiography

## Abstract

**Aims:**

We aimed to investigate and present cases of perimyocarditis and pericarditis verified by cardiovascular resonance (CMR) imaging in patients with a strong temporal association to SARS-CoV-2 vaccination. We sought to describe the clinical presentation including coronary artery angiography, CMR, transthoracic echocardiography, blood samples, electrocardiography, and symptoms.

**Methods:**

We included 10 patients admitted with chest pain shortly after vaccination for SARS-CoV-2, who were diagnosed with pericarditis or perimyocarditis by CMR. We reviewed the CMR, echocardiography, electrocardiography, blood samples, coronary artery angiography, vital signs and medical history. The updated Lake Louise Criteria were used to determine the diagnosis by CMR.

**Results:**

Eight patients had perimyocarditis and two patients had pericarditis. The mean age was 22 ± 5 years (range 16 to 31 years), 90% were male. The median time from vaccination to hospital admission was 4 days (range 2 to 28 days). Admissions were seen after vaccination with three different SARS-CoV-2 vaccine manufacturers. Nine Patients had ST-elevation on the initial electrocardiography. Peak troponins varied from 357 to 23,547 ng/l, with a median of 4,304 ng/l. Two patients had an LVEF <50% on echocardiography and four patients had left ventricular global longitudinal strain values <18%. CMR revealed preserved left ventricular ejection fraction (LVEF), although one patient had decreased LVEF on CMR. The T1 and T2 mapping values were increased in all patients. Of the 8 patients with perimyocarditis, all patients had signs of myocardial injury in the lateral segments of the left ventricle.

**Conclusions:**

This case series of 10 patients supports the emerging evidence of an association between vaccination for SARS-CoV-2 and perimyocarditis and pericarditis, especially in young males. The temporal association was seen after vaccines from three different manufacturers. Imaging data from echocardiography and CMR displayed normal to mildly impaired cardiac function, usually with a mild disease course.

## Introduction

Perimyocarditis is an inflammation of the pericardium and the underlying myocardium, most commonly of viral etiology (1). Non-infectious etiologies have been reported, although these are less common. Recently perimyocarditis has been reported as a possible side-effect to severe acute respiratory syndrome coronavirus 2 (SARS-CoV-2) vaccines. Emerging evidence indicates an increased incidence of perimyocarditis after vaccination for SARS-CoV-2, particularly when vaccinated with a messenger RNA (mRNA) vaccine. Population-based data from Israel reported an increased incidence of myocarditis following mRNA vaccination, that in particular seems to affect young men (2–4). However, the association between the different vaccines available and pericarditis, myocarditis or perimyocarditis has not been definitively established, as the causality is based solely on the temporal association. To investigate this possible association, we sought to elaborate the presentation of these patients by systematic clinical assessment, blood samples and detailed cardiac imaging.

When the SARS-CoV-2 vaccines were introduced for a world-wide vaccination campaign in adults, the phase 3 trials of the vaccines revealed an acceptable safety profile (5–7). The reported side effects were generally mild to moderate, and symptoms following all 4 European Union (EU) approved vaccines usually disappeared within a few days. However, when introducing a vaccination campaign in all adults in the EU, the risk of unidentified side effects is likely to increase considerably. Therefore, the use of passive surveillance systems such as the EU EudraVigilance database of suspected adverse drug reaction reports is essential for continuous safety profile assessment of the vaccines (8). As of October 26^th^, 2021, 3,581 cases of pericarditis (Pfizer-Biontech 2,667; Moderna 816; Janssen 98) and 4,931 cases of myocarditis (Pfizer-Biontech 3,292; Moderna 1,540; Janssen 99) has been reported to the EudraVigilance database. The EudraVigilance report system is limited by the passive nature of reporting adverse events and does not provide detailed descriptions of the clinical presentation, severity and course of the disease. Although a few case reports have been presented (9–11), more detailed information regarding the clinical presentation, biochemistry, and cardiac imaging findings of the presentation of perimyocarditis in patients with a temporal association to vaccination for SARS-CoV-2 is needed.

In this study we present 10 cases of perimyocarditis or pericarditis verified by cardiovascular magnetic resonance imaging (CMR) in patients with a strong temporal association to SARS-CoV-2 vaccination. We sought to provide a full clinical presentation including coronary artery angiography when available, CMR, transthoracic echocardiography (TTE), blood samples, electrocardiography (ECG), and clinical symptoms.

## Methods

During the period of June 1^st^ to October 1^st^ 2021, a total of 22 patients from the central Denmark Region were diagnosed with perimyocarditis, 2 with myocarditis and 9 with pericarditis by CMR at Aarhus University Hospital, Denmark. A total of 10 patients who fulfilled the diagnostic criteria of perimyocarditis or pericarditis were included in the study since they presented with symptoms <28 days after receiving the vaccine and had no previous infection with SARS-CoV-2. All 10 patients were hospitalized due to chest pain or functional dyspnea. All patients, or their legally authorized representative, provided written consent for their participation. This study complies with the declaration of Helsinki and was exempt from formal approval by the institutional review board due to the observational nature of the study.

### Diagnostic criteria

The diagnosis of perimyocarditis was established based on relevant clinical symptoms, ECG findings, troponin measurement, echocardiographic examination, and the updated Lake Louise Criteria by CMR (12). The CMR findings used for determining diagnosis were the combination of myocardial oedema together with other CMR markers of inflammatory myocardial injury. Thus, the CMR diagnosis of perimyocarditis was based on at least one T2-based criterion (increase of myocardial T2 relaxation time or an increased signal intensity in T2-weighted images) with at least one T1-based criterion (increased myocardial T1, extracellular volume, or late gadolinium enhancement). The diagnosis of pericarditis was established with early and late gadolinium enhancement of the pericardium without signs of myocardial oedema or injury assessed by T1 and T2-mapping. No endomyocardial biopsies were obtained in these patients.

### Transthoracic echocardiography

All patients underwent echocardiographic assessment according to current guidelines (13), performed using the GE Vivid E9 or E95 (GE healthcare, Horten, Norway) with a standard phased array transducer. Offline analysis was performed using Echopac version 203 (PC SW-only, GE Healthcare, Milwaukee, Wisconsin, USA). Echocardiography was performed during the initial admission.

Left ventricular (LV) ejection fraction (LV EF) was calculated using the modified Simpsons biplane method of disks. LV global longitudinal strain (GLS) was estimated using 2-dimensional cine-loops with a frame rate >55 frames/s measured in the three standard apical views. The automated function automatically traced the endocardial border and was manually adjusted as appropriate. The software automatically generated a 17-segment bullseye plot with the presented values being an average of all 17 segments. Peak early (E) and late (A) diastolic mitral inflow velocity were measured using pulsed-wave Doppler, placed at the tip of the mitral valve leaflets in the apical 4-chamber view. Lateral mitral annular diastolic velocity (e') was measured using tissue Doppler images in the apical 4-chamber view.

### Cardiac magnetic resonance imaging

A standard CMR protocol was performed using a Philips Achieva dStream 1.5 T whole body MR scanner (Philips Medical Systems, Best, Netherlands). The clinical protocol to assess aortic flow and ventricular volumes and mass was performed during end expiratory breath hold according to consensus guidelines (14). Tissue characterization was performed with T1 pre and post contrast and T2 mapping to visualize myocardial injury and oedema, respectively. A T2-weighted short tau inversion recovery sequence was used to visualize edema (15).

Late Gadolinium Enhancement (LGE) imaging was performed 10–15 min after intravenous administration of 0.1 mmol/kg gadobutrol (Gadovist, Bayer HealthCare, Berlin, Germany). Cardiac mass, volumes and strain of the left and right ventricle were analyzed using Segment version 3.2 (Medviso AB, Lund, Sweden). The extent of affected myocardium was determined semi-automatically by software-based counting of hyperenhanced pixels in the myocardium on each of the short-axis LGE images. Hyperenhanced pixels were defined as those with image intensities of 5 SDs above the mean of image intensities in a remote myocardial region. The relative affected volume was then calculated as a percentage of LV volume, as the sum of hyperenhanced pixels from each of the short-axis images divided by the total number of pixels within the LV myocardium.

LV measurements were obtained using automated 3-dimensional (3D) segmentation in the short axis view with the possibility of subsequent manual adjustment. The right ventricular (RV) measurements were obtained using a semi-automated 3D tool and were subsequently adjusted manually as appropriate. Radial strain of the left ventricle was estimated by tracing the endo- and epicardial borders in diastole and systole. LV longitudinal strain was estimated in 3D by tracing the borders of the left ventricle in the 2-, 3- and 4-chamber views. RV strain was assessed in the 4-chamber view. We have previously published CMR derived strain values of healthy volunteers and patients with perimyocarditis using the same CMR scanner and the same analysis tool (16). We found CMR derived longitudinal strain values of 15.1 ± 1.2 in healthy volunteers with a mean age of 63 years and longitudinal strain values of 13.7 ± 3.0 in patients with perimyocarditis with a mean age of 30 years.

### Blood samples and electrocardiograms

All blood samples and ECGs were obtained on clinical indication. Troponins were measured several times in all patients during hospitalization, with the peak troponins being reported in this study. A troponin I (TnI) <47 ng/l was considered normal.

All patients had routine viral serology performed to rule out parotitis, Borrelia Burgdorferi, Epstein Barr, influenza-virus, parvovirus, adenovirus, cytomegalovirus and a blood-culture to exclude other common etiologies of perimyocarditis.

### Coronary artery imaging

Coronary computed tomography angiography (CCTA) and invasive coronary angiogram (CAG) were performed on clinical indication if the treating physicians deemed it necessary to exclude acute ischemic pathology as the cause of the clinical presentation.

### Incidence of pericarditis and myocarditis

The incidence of pericarditis and myocarditis in the Central Denmark Region was estimated using the Business Intelligence (BI) database in the Central Denmark Region. The ICD-10 diagnosis codes used for the BI database were DI309 (acute pericarditis) and DI409 (acute myocarditis). Unique patients with either of the two diagnoses living in the central Denmark Region between the ages 15 and 35 years were drawn from the database from 1^st^ of June to 31^st^ of September in 2019, 2020 and 2021. Counts of citizens between 15 and 35 years of age living in the Central Denmark Region were extracted from the Statistics Denmark database in the 3^rd^ quarter of 2019, 2020, and 2021.

## Results

### Patient characteristics

**Figure 1:**
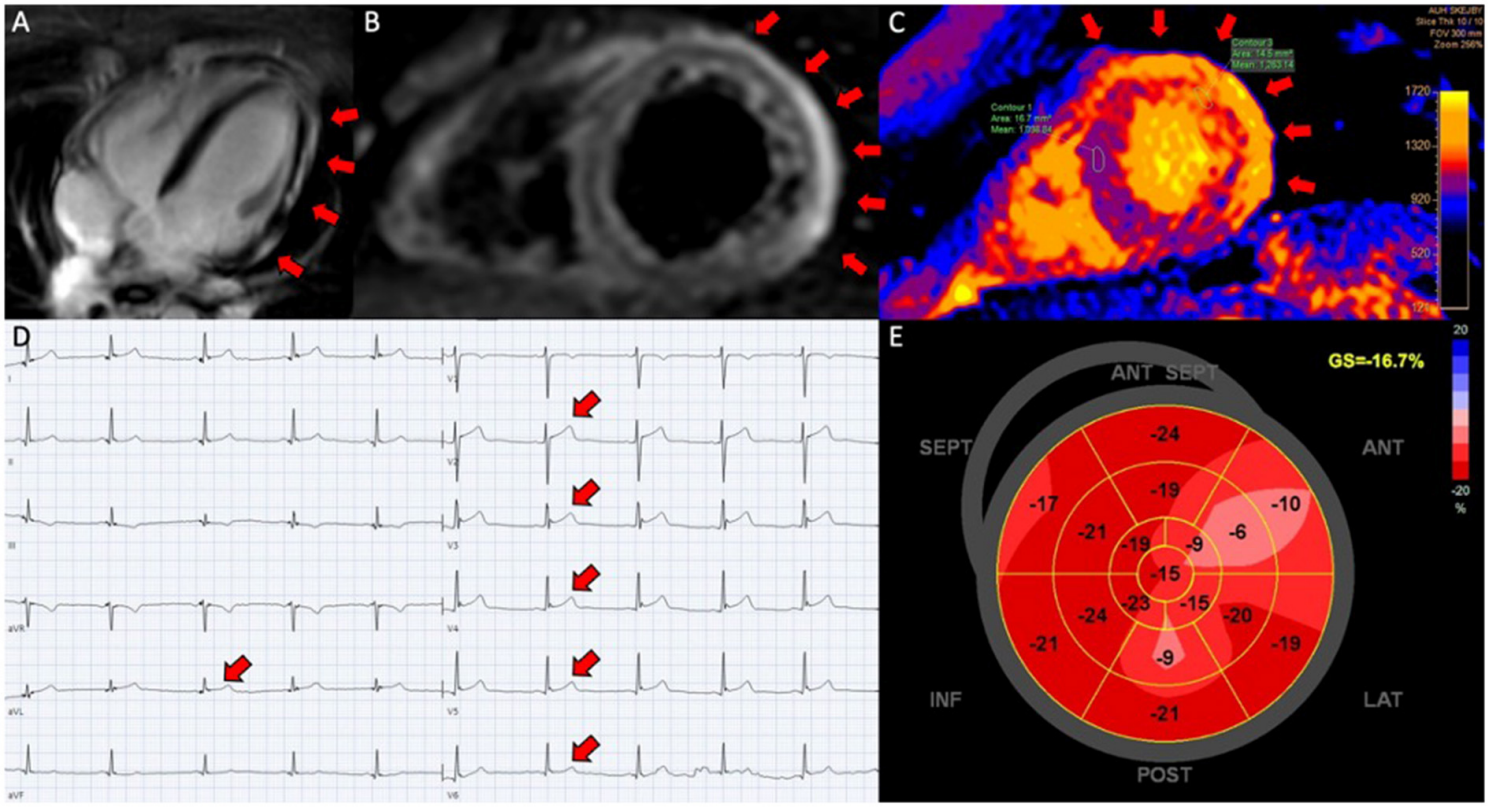
Perimyocarditis in a patient (case 3). **(A)** Late gadolinium enhancement in the epicardium at the lateral segments in 4-chamber view; **(B)** CMR, short axis T2-weigthed image showing hyper-intensive signal in the epicardium along the lateral segments indicative of oedema; **(C)** T1-map in short axis showing increased T1 values up to 1,191 ms at the lateral segments reflecting myocardial injury; **(D)** electrocardiogram showing ST-elevations in lead V2-V6 and aVL; **(E)** 17-segment plot of global longitudinal strain on echocardiography with decreased values antero-laterally.

**Figure 2:**
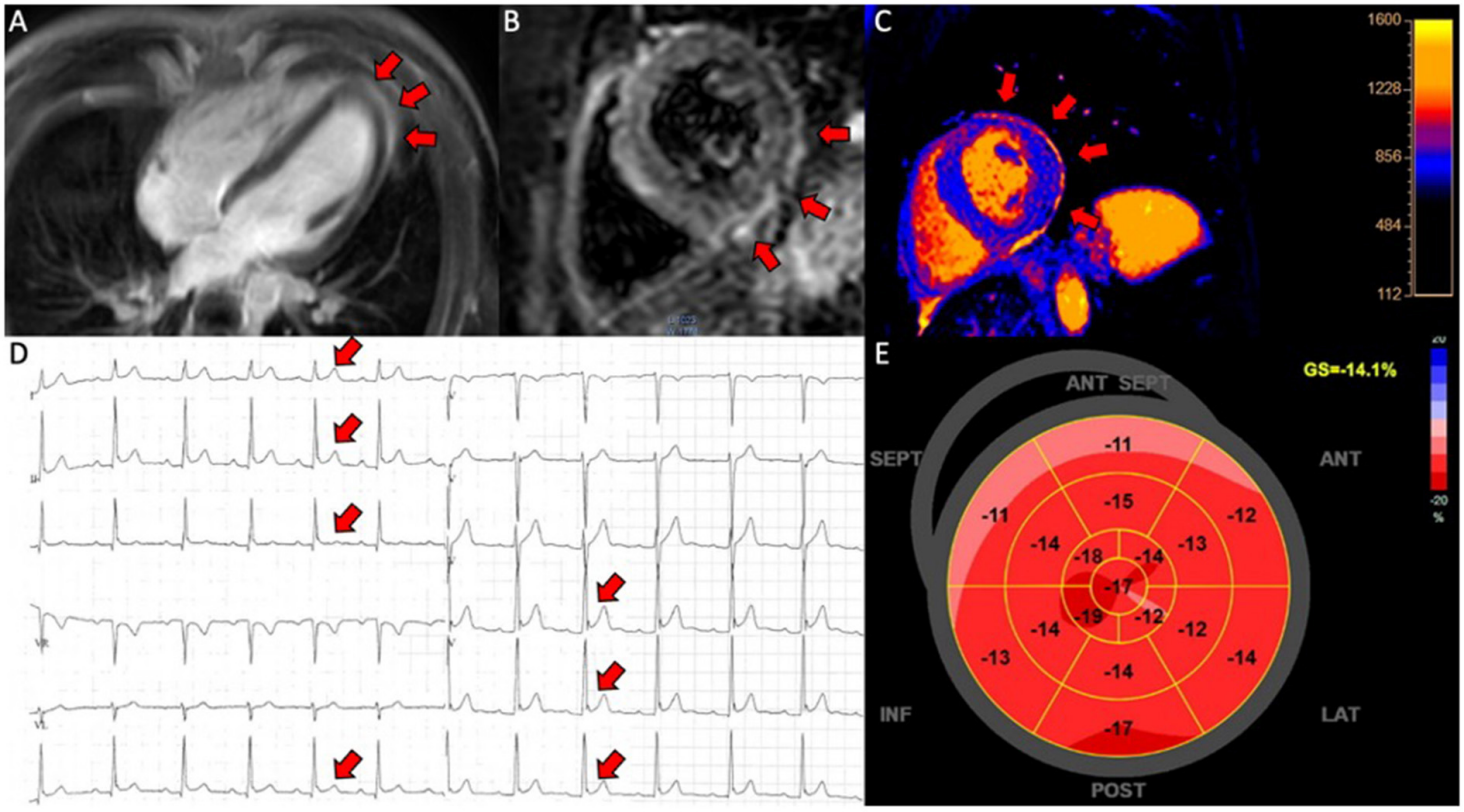
Pericarditis in a patient (case 9). **(A)** CMR, late gadolinium enhancement in the pericardium in 4-chamber view; **(B)** CMR, short axis T2-weigthed image showing hyper-intensive signal in the pericardium indicative of oedema; **(C)** CMR, T1-map in short axis showing increased T1 values up to 1,231 ms in the pericardium; **(D)** electrocardiogram showing ST-elevations in lead I, II, III, aVF and V4-V6; **(E)** 17-segment plot of global longitudinal strain on echocardiography with decreased values in the basal segments anteriorly.

Ten patients were included with a mean age of 22 ± 5 years (range 16–31 years). All patients were referred for hospital admission due to chest pain and/or dyspnea. Nine patients (90 %) were male. Seven patients (78 %) received the BNT162b2 mRNA vaccine from Pfizer-Biontech and two (20 %) received the mRNA1273 vaccine from Moderna and one (10 %) received the Ad26.COV2.S vaccine from Janssen. Four patients (44 %) developed perimyocarditis after the 1^st^ dose and five (56 %) developed symptoms after the 2^nd^ dose. One patient developed pericarditis after the 1^st^ dose and one developed pericarditis after the 2^nd^ dose. No patients had previously been infected with SARS-CoV-2. Eight patients were considered to have perimyocarditis and two patient were diagnosed with pericarditis. An example of perimyocarditis is presented in Figure 1 and an example of pericarditis is presented in Figure 2. The median time from receiving the vaccine to hospital admission was 4 days [interquartile range (IQR) 3 to 17 days] with 2 days being the shortest time and 28 days being the longest. Seventy percentage of the patients were admitted to the hospital within 6 days after receiving the vaccine dose. The mean duration of hospitalization was 3.6 ± 1.5 days. One patient had previously been diagnosed with perimyocarditis 5 years prior to admission. One patient had recurrent chest pain leading to rehospitalization 2 months after the initial admission and received intensified anti-inflammatory treatment. Two patients (20%) were diagnosed with attention deficit hyperactivity disorder (ADHD) and one patient was diagnosed with attention deficit disorder (ADD). All patients with ADHD or ADD were previously or currently under treatment with methylphenidate, with all three patients having received methylphenidate for at least 3 years.

### Symptoms, electrocardiography, and biochemistry

All patients presented with chest pain. Palpitations and dyspnea were lesser common symptoms at presentation (*n* = 2 and *n* = 3, respectively). Demography, vital signs and electrocardiograms are presented in Table 1. Seven of the patients had fever (temperature ≥ 37.5 degree Celsius) at the time of admission.

**Table 1:** Demography, vital signs, and electrocardiography.

**Case number**										
	**1**	**2**	**3**	**4**	**5**	**6**	**7**	**8**	**9**	**10**
Age, years	16	24	22	21	23	16	28	17	24	31
Sex	Male	Female	Male	Male	Male	Male	Male	Male	Male	Male
Vaccine (dose)	Pfizer (2nd)	Moderna (1st)	Pfizer (1st)	Pfizer (2nd)	Pfizer (2nd)	Pfizer (2nd)	Moderna (1st)	Pfizer (1st)	Jannsen (1st)	Pfizer (2nd)
Diagnosis	PM	PM	PM	PM	P	PM	PM	PM	P	PM
Treatment	–	–	–	Col.	–	–	–	Col. Steroid	–	Morphine
Days from vaccination to adm.	3	21	28	17	2	2	6	5	3	3
Duration of hospital adm., days	5	5	5	4	3	3	5	4	1	1
Previous SARS-CoV-2 (days prior to vax)	No	No	No	No	No	No	No	No	No	No
**Vital signs on admission**										
Temp., C	36.6	38.9	38.5	37.6	37.3	37.4	37.5	36.9	37.9	38
Heartrate beats/min.	89	125	86	97	86	113	97	57	78	84
BP, mmHG	122/75	111/68	102/62	121/80	151/86	106/66	115/64	120/57	142/90	131/80
RR	16	24	14	16	12	16	28	20	14	18
**Electrocardiogram on admission**										
Rhytm	SR	SR	SR	SR	SR	SR	SR	SR	SR	SR
Leads with ST-elevation	I, II, V4-V6	Normal	V2-V6, aVL	I, II, aVF, V2-V6	I, II, III, aVF, V2-V4	I, II, III, aVF, V5-V6	I, aVF, V2-V6	II, III, aVF,	I, II, III, aVF, V4-V6	II, III, aVF
**Biochemistry**										
TnI - peak	357 (TnT)	2,535	23,547	10,748		1,512 (TnT)	13,007	3,653		4,954
CRP	7	271	99	88	72	52	68	40	<4	33

Adm., admission; BP, blood pressure; Col, Colchicin; CRP, c-reactive protein; min, minute; P, pericarditis; PM, perimyocarditis; RR, respiratory rate; SR, sinus rhytm; temp, temperature; TnI, troponin I; TnT, troponin T; vax, vaccination.

Nine (90%) of the patients had ST segment elevation on ECG. Peak cardiac Troponin I varied from 357 to 23,547 ng/l with a median of 4,304 ng/l (IQR 2,024 to 11,878 ng/l) for patients with perimyocarditis. The median CRP was 60 mg/l (IQR 33 to 88 mg/l) at admission and the mean leucocyte count was 9.2 ± 2.5 ^*^ 10^9^/l.

### Echocardiography

Results from echocardiography and CMR are presented in Table 2. The mean LV ejection fraction (EF) was 55 ± 7%. Two patients (20%) had an LVEF <50%. The mean LV GLS was−17.2 ± 2 %, with *n* = 4 (40%) having an absolute LV GLS < 18%. The mean E/A ratio was 1.6 ± 0.3 and the mean E/e' ratio was 5.5 ± 1.2. No patients had signs of pericardial effusion on echocardiography.

**Table 2:** Cardiovascular magnetic resonance and echocardiographic parameters.

	**1**	**2**	**3**	**4**	**5**	**6**	**7**	**8**	**9**	**10**
**Cardiovascular magnetic resonance**										
Days from admission to CMR	14	11	7	2	7	7	1	17	55	3
LV EF, %	54	63	63	48	56	51	52	60	58	61
LV EDV, ml	201	168	62.3	154.5	133	175.5	172.1	192.6	165.5	112.4
LV ESV, ml	103	62.1	23.0	80.3	58.4	86.5	81.8	77.2	68.8	43.4
Strain radial, %	−15.0	−19.3	−21.3	−12.9	−19.5	−17.1	−16.7	−20.1	−18.6	−21.7
Strain, longitudinal, %	−13.2	−17.0	−18.0	−11.7	−15.3	−17.2	−15.3	−19.3	−14.4	−17.2
RV EF, %	60	64	67	58	65	62	48	59	62	64
RV strain, %	−18.1	−19.0	−20.6	−14.6	−15.2	−19.4	−19.4	−19.5	−20.3	−21.7
LV mass, g	133.2	88.4	62.5	125.2	101.2	77.4	109.2	113.8	118.5	102.9
Median T1	1,029	1,099	1,020	1,143	999	1,074	1,058	1,098	1,020	1,098
T1 Min	997	1,062	980	1,069	959	1,026	1,028	1,062	980	1,052
T1 max	1,067	1,168	1,191	1,332	1,023	1,120	1,119	1,141	1,051	1,238
Median T2	48	54	51	55	51	57	52	53	51	55
T2 min/max	42/56	49/62	46/70	41/63	45/56	51/64	47/60	50/63	46/61	46/67
LGE, %	1.7	0.6	7.6	15.8	1.2	1.3	14.7	0.3		0.6
**Echocardiography at admission**										
LV EF, %	52	55	60	43	55	45	60	65	60	53
LV GLS, %	−19.8	−19.6	−16.7	−13.6	−16.4	−18.0	−18.1	-	−14.1	−18.2
E/A-ratio	1.59	1.29	2.21	1.52	1.63	1.58	1.58	-	1.05	2.16
E/e'-ratio	7.53	4.35	7.73	6.06	4.89	5.54	5.54	-	3.86	5.90
**CAG/HCT**										
	-	-	CCTA	-	CCTA	-	ICA	-		ICA

ICA, invasive coronary angiogram; CMR, cardiovascular magnetic resonance; EDV, end diastolic volume; EF, ejection fraction; ESV, end systolic volume; GLS, global longitudinal strain; CCTA, coronary computed tomography angiography; LGE, late gadolinium enhancement; LV, left ventricle; RV, right ventricle.

### Cardiac magnetic resonance imaging

The median time from admission to CMR was 7 days (IQR 3 to 14 days). The mean LVEF was 57 ± 5 %, the mean LV longitudinal strain was−15.9 ± 2 % and the mean radial LV strain was−18.2 ± 2.8 %. The mean RV EF was 61 ± 5 % and the mean longitudinal strain of the right ventricle was−18.8 ± 2 %. The median native T1 mapping value was 1,066 (IQR 1,020 to 1,098) [a native T1 value of 1,003 ± 46 was considered normal (17)]. The median T2 mapping value was 53 (IQR 51 to 55) and the maximum and minimum T2 values are presented for each patient in Table 2. The normal range of myocardial T2 relaxation times has been reported to be within 50–62 ms (17). The most common localization of myocardial injury, assessed using LGE, was in the lateral segments of the LV (*n* = 10, 83%), although one patient with pericarditis had oedema anteriorly in the LV and the other patient with pericarditis had oedema in the lateral segments of the left ventricle.

### Incidence of pericarditis and perimyocarditis in the central denmark region

From June 1^st^ to September 31^st^ in 2019, 29 (of 361.849) patients between 15 and 35 years of age were diagnosed with pericarditis and/or myocarditis in the Central Denmark Region. In 2020 the number was 25 (of 363.611) and in 2021 the number was 38 (of 367.212) patients between 15 and 35 years who were diagnosed with pericarditis and/or myocarditis between 1^st^ of June and 31^st^ of September.

## Discussion

In this study we present patients diagnosed with perimyocarditis and pericarditis who all had a temporal association to three different types of vaccines for SARS-CoV-2. All patients had relatively mild presentation of disease, with short symptom duration and hospitalization ranging from 1 to 5 days.

### Clinical presentation

This series of cases supports the emerging evidence suggesting that the typical patient suffering from perimyocarditis following vaccination for SARS-CoV-2 is predominantly male, with an age between 15 and 35 years, presenting with chest pain. ECG changes with ST elevation is often identified on admission, however the ECG may be normal in some cases. Blood samples will often show increased cardiac biomarkers such as troponins if there is myocardial involvement but can be normal if the inflammation is confined to the pericardium. Echocardiography will often reveal preserved systolic and diastolic function, although some patients may present with slightly decreased longitudinal LV systolic function (18). CMR will show late gadolinium enhancement in the pericardium and in the majority of patients in the subepicardial myocardium, reflecting ongoing tissue inflammation. Myocardial edema depicted by increased myocardial T2 values is present in those with perimyocarditis. The inflammation visualized by CMR is often located in the lateral segments of the LV (18).

### Pathophysiology

The pathophysiological mechanisms behind the potential association between the immune response from SARS-CoV-2 vaccines and perimyocarditis are poorly understood. Myocarditis after vaccination is not a new phenomenon and has previously been reported after smallpox- and influenza vaccination (19). We know from the vaccine trials, that younger adults develop a stronger immune response to the vaccines which provide a stronger protection against SARS-CoV-2, however this may simultaneously increase their risk of adverse immunological side effects. Molecular mimicry, whereby there is antigenic cross reactivity between the cardiac tissue and the resulting antibodies from the vaccines, has been suggested as a potential mechanism of action. Vojdani and Kharrazian (20) have investigated the reaction between SARS-CoV-2 spike protein antibodies and various tissue proteins. Of particular interest, cross reactivity was found to be strong between SARS-CoV-2 spike protein antibodies and transglutaminase 2, alpha-myosin and collagen which are proteins expressed in the cardiac atria, ventricles and the pericardium (21–23). This cross reactivity could lead to an autoimmune reaction against myocytes and the pericardium, which would cause the inflammation seen in myocarditis, pericarditis and perimyocarditis. Furthermore, this mechanism would explain why there are more reports of myocarditis and perimyocarditis after the 2^nd^ dose, and primarily with mRNA vaccines, as these are likely to trigger a larger immune response. This is particularly the case in young adults.

### Incidence of myocarditis after vaccination

During the 4 month period from 1^st^ of June to 31^st^ of September we found an incidence of pericarditis and/or myocarditis of 1 in 12.478 in citizens aged 15–35 years in the Central Denmark Region in 2019, 1 in 14.544 in 2020 and 1 in 9.663 in 2021, based on reports from the regional database. Even though register data should be assessed with caution, these data suggest an overall increased incidence of pericarditis and myocarditis in citizens between 15 and 35 years of age in the period where the vaccination program was initiated in this age-group. Retrospective studies report increased incidences of myocarditis in young males vaccinated with the BNT162b mRNA vaccine from Pfizer-Biontech. Witberg et al. (2) report an incidence of 10.69 cases of myocarditis after vaccination per 100.000 persons in male patients between 16 and 29 years of age. Furthermore, Barda et al. (4) reported a risk difference of 13.73 per 100.000 persons between the 1^st^ and 2^nd^ dose in males between 16 and 19 years.

This risk needs to be balanced against the incidences of myocarditis after infection with SARS-CoV-2. The overall risk ratio of myocarditis after vaccination for SARS-CoV-2 has been estimated to be 3.24 (risk difference 2.7 events per 100.000 persons) across all ages and sex, compared to a matched unvaccinated control group (4). The risk of myocarditis after infection with SARS-CoV-2 was estimated to a risk ratio of 18.28 (risk difference 11.0 events per 100.000 person years) (4). This retrospective analysis suggests that there is an increased risk of myocarditis after vaccination for SARS-CoV-2 with mRNA vaccines, but the risk of myocarditis seems substantially larger after infection with SARS-CoV-2. However, these data are limited by their retrospective nature, with a substantial risk of bias. Furthermore, in these study reports authors have not systematically used CMR or endomyocardial biopsies to validate the diagnosis of myocarditis. The risk profile of the SARS-CoV-2 vaccines still favors vaccination, even in young male individuals, as the risk of perimyocarditis is relatively modest and with a mild disease course, especially when compared to the risk profile of infection with SARS-CoV-2. The remaining question clinicians face is, whether to recommend another dose, either the 2^nd^ or 3^rd^ dose of the vaccines, in individuals who have already suffered from perimyocarditis shortly after a previous dose of the vaccine.

### Short- and long-term effects

The short-term effects of perimyocarditis following vaccination for SARS-CoV-2 seem to be relatively mild even though all patients experienced chest pain requiring hospitalization. The disease often resulted in a relatively short hospitalization and rapid clinical improvement. Discharge occurred within seven days with relatively modest effects on echocardiographic and CMR parameters. The most common complaint from the patients after discharge was the advised abstinence from intense physical training. However, currently the long-term prognosis of perimyocarditis in these young patients with SARS-CoV-2 vaccine related myocarditis remains unknown. One case report demonstrated full functional recovery at 3 months in a young patient with SARS-CoV-2 vaccine related myocarditis (24). This is in accordance with studies showing that in approximately 50% of patients with myocarditis there is resolution within 2–4 weeks, however the prognosis depends on the etiology (25, 26).

## Limitations

This study has several limitations. The presumed association between vaccination and perimyocarditis is based on the temporal association, and not validation with viral mRNA diagnostics using endomyocardial biopsies. However, the findings are in line with other case reports and studies suggesting the possible link, and due to the relatively mild nature of the disease courses, there were no indications for endomyocardial biopsies. We do not have long-term follow-up in these patients yet, as younger citizens (age below 35 years) were offered vaccination from May-July 2021 in Denmark, but a follow-up program is planned. We aimed to perform CMR within 7 days after admission, but in one case the CMRI was performed after 55 days. We used data from a single center, and as such the number of patients is limited. However, this study is the largest detailed report of cases, and the results are in line with data presented in large retrospective studies. Since we could only diagnose patients who were admitted to the hospital, it is possible that the true incidence of perimyocarditis following vaccination for SARS-CoV-2 is higher. It could be speculated that subclinical cases have not been diagnosed.

## Conclusion

This case series of 10 patients supports the emerging evidence of an association between vaccination for SARS-CoV-2 and perimyocarditis, especially in young males. The temporal association was seen after vaccines from three different manufacturers, both mRNA vaccines and vector-based vaccines. The main clinical symptom was chest pain leading to hospitalization. Imaging data from echocardiography and CMR displayed normal to mildly impaired cardiac function. Further studies are needed to investigate the long-term clinical and prognostic consequences of this assumed vaccine related entity.

## Data availability statement

The raw data supporting the conclusions of this article will be made available by the authors, without undue reservation.

## Ethics statement

Ethical review and approval was not required for the study on human participants in accordance with the local legislation and institutional requirements. Written informed consent to participate in this study was provided by the participants' legal guardian/next of kin. Written informed consent was obtained from the individual(s), and minor(s)' legal guardian/next of kin, for the publication of any potentially identifiable images or data included in this article.

## Author contributions

AP, SP, and WK planned the project and wrote the first manuscript draft. AP, GW, CF, MJ, WK, and SP contributed to data collection. BL and LØ provided critical feedback and helped shape the research and analysis. All authors contributed to the article and approved the submitted version.

## Funding

This work was supported by the Novo Nordisk Foundation (Grant No. NNF21OC0066984). WK was funded by the Health Research Fund of Central Denmark Region.

## Conflict of interest

The authors declare that the research was conducted in the absence of any commercial or financial relationships that could be construed as a potential conflict of interest.

## Publisher's note

All claims expressed in this article are solely those of the authors and do not necessarily represent those of their affiliated organizations, or those of the publisher, the editors and the reviewers. Any product that may be evaluated in this article, or claim that may be made by its manufacturer, is not guaranteed or endorsed by the publisher.
